# Efficacy and Safety of Pharmacokinetically-Driven Dosing of Mycophenolate Mofetil for the Treatment of Pediatric Proliferative Lupus Nephritis–A Double-Blind Placebo Controlled Clinical Trial (The Pediatric Lupus Nephritis Mycophenolate Mofetil Study)

**Published:** 2024-05-13

**Authors:** Anna Carmela P Sagcal-Gironella, Angela Merritt, Tomoyuki Mizuno, Vikas R Dharnidharka, Joseph McDonald, Marietta DeGuzman, Dawn Wahezi, Beatrice Goilav, Karen Onel, Susan Kim, Ellen Cody, Eveline Y Wu, Laura Cannon, Kristen Hayward, Daryl M Okamura, Pooja N Patel, Larry A Greenbaum, Kelly A Rouster-Stevens, Jennifer C Cooper, Natasha M Ruth, Stacy Ardoin, Kathryn Cook, R Ezequiel Borgia, Aimee Hersh, Bin Huang, Prasad Devarajan, Hermine Brunner

**Affiliations:** 1 Division of Pediatric Rheumatology, Hackensack University Medical Center, Hackensack, New Jersey, USA; 2 Department of Pediatrics, Hackensack Meridian School of Medicine, Nutley, New Jersey, USA; 3 Division of Rheumatology, Cincinnati Children’s Hospital Medical Center, Cincinnati, Ohio, USA; 4 Division of Translational and Clinical Pharmacology, Cincinnati Children’s Hospital Medical Center, Cincinnati, Ohio, USA; 5 Department of Pediatrics, University of Cincinnati College of Medicine, Cincinnati, Ohio, USA; 6 Department of Pediatric Nephrology, Washington University School of Medicine, Saint Louis, Missouri, USA; 7 Department of Pediatrics, University of Chicago, Chicago, Illinois, USA; 8 Department of Pediatrics, Baylor College of Medicine, Houston, Texas, USA; 9 Department of Pediatric Rheumatology, Children’s Hospital at Montefiore, Albert Einstein College of Medicine, Bronx, New York, USA; 10 Pediatric Nephrology, The Children’s Hospital at Montefiore, Albert Einstein College of Medicine, Bronx, New York, USA; 11 Department of Pediatric Rheumatology, Hospital for Special Surgery, Weill Cornell Medicine, New York, New York, USA; 12 Department of Rheumatology, University of California, San Francisco, California, USA; 13 Department of Pediatric Nephrology, Medical College of Wisconsin, Milwaukee, Wisconsin, USA; 14 Department of Pediatric Rheumatology, University of North Carolina, Chapel Hill, North Carolina, USA; 15 Department of Pediatric Rheumatology, University of Washington, Seattle, Washington, USA; 16 Department of Pediatric Nephrology, University of Washington, Seattle, Washington, USA; 17 Depatrment of Pediatric Rheumatology, Ann & Robert H. Lurie Children’s Hospital of Chicago, Northwestern University, Chicago, Illinois, USA; 18 Department of Pediatric Nephrology, Emory University and Children’s Healthcare of Atlanta, Atlanta, Georgia, USA; 19 Department of Pediatrics, Emory University and Children’s Healthcare of Atlanta, Atlanta, Georgia, USA; 20 Department of Pediatric Rheumatology, University of Colorado, Denver, Colorado, USA; 21 Department of Pediatric Rheumatology, Medical University of South Carolina, Charleston, South Carolina, USA; 22 Abigail Wexner Research Institute, Nationwide Children’s Hospital, Columbus, Ohio, USA; 23 Division of Rheumatology, Akron Children’s, Akron, Ohio, USA; 24 Department of Pediatric Allergy, Immunology and Rheumatology, UH Rainbow Babies & Children’s Hospital, Cleveland, Ohio, USA; 25 Department of Pediatrics, Immunology and Rheumatology, University of Utah, Salt Lake City, Utah, USA; 26 Department of Pediatrics, University of Cincinnati College of Medicine and Cincinnati Children’s Hospital Medical Center, Cincinnati, Ohio, USA; 27 Department of Nephrology and Hypertension, Cincinnati Children’s Hospital Medical Center, Cincinnati, Ohio, USA

**Keywords:** Pediatric, Lupus, Childhood-onset, SLE, Lupus nephritis, Mycophenolate mofetil, Pharmacokinetics

## Abstract

**Background:**

The safety and efficacy of mycophenolate mofetil (MMF) for lupus nephritis (LN) treatment is established in adults and in some children. MMF is rapidly converted to the biologically active metabolite mycophenolic acid (MPA) whose pharmacokinetics (PK) is characterized by large inter- and intra-individual variability.

**Methods/Design:**

This randomized, double-blind, active comparator, controlled clinical trial of pediatric subjects with proliferative LN compares pharmacokinetically-guided precision-dosing of MMF (MMF_PK_, i.e. the dose is adjusted to the target area under the concentration-time curve (AUC_0–12h_) of MPA ≥ 60–70 mg*h/L) and MMF dosed per body surface area (MMF_BSA_, i.e. MMF dosed 600 mg/m^2^ body surface area), with MMF dosage taken about 12 hours apart. At baseline, subjects are randomized 1:1 to receive blinded treatment with MMF_PK_ or MMF_BSA_ for up to 53 weeks. The primary outcome is partial clinical remission of LN (partial renal response, PRR) at week 26, and the major secondary outcome is complete renal response (CRR) at week 26. Subjects in the MMF_BSA_ arm with PRR at week 26 will receive MMF_PK_ from week 26 onwards, while subjects with CRR will continue MMF_BSA_ or MMF_PK_ treatment until week 53. Subjects who achieve PRR at week 26 are discontinued from study intervention.

**Discussion:**

The Pediatric Lupus Nephritis Mycophenolate Mofetil (PLUMM) study will provide a thorough evaluation of the PK of MMF in pediatric LN patients, yielding a head-to-head comparison of MMF_BSA_ and MMF_PK_ for both safety and efficacy. This study has the potential to change current treatment recommendations for pediatric LN, thereby significantly impacting childhood-onset SLE (cSLE) disease prognosis and current clinical practice.

## INTRODUCTION

Systemic lupus erythematosus (SLE) is a multi-organ autoimmune disease with relatively increasing mortality that often targets young women and children of U.S. minorities [1–3]. Childhood-onset SLE (cSLE) has similar manifestations as lupus in adults, but childhood-onset disease is accompanied by more severe multi-organ involvement, including LN in up to 80% of affected children [4–6].

Although the use of cyclophosphamide (CYC) has long been regarded the standard of care in proliferative LN [7], MMF is similarly accepted as a cornerstone of LN therapy [8], with a more favorable side-effect profile compared with CYC [9]. Off-label use for the treatment of LN in Europe and the U.S. is supported by recommendations of the American College of Rheumatology (ACR) [10] and the European League against Rheumatism (EULAR) [11]. The safety and efficacy of MMF in LN have been tested in adults and in some pediatric subjects. However, since the optimal MMF dosing to achieve improvement in LN in cSLE is not well-established [12], the pediatric renal transplant dosing regimen of MMF based on weight or body surface area (BSA) has been adopted instead.

**Figure 1:**
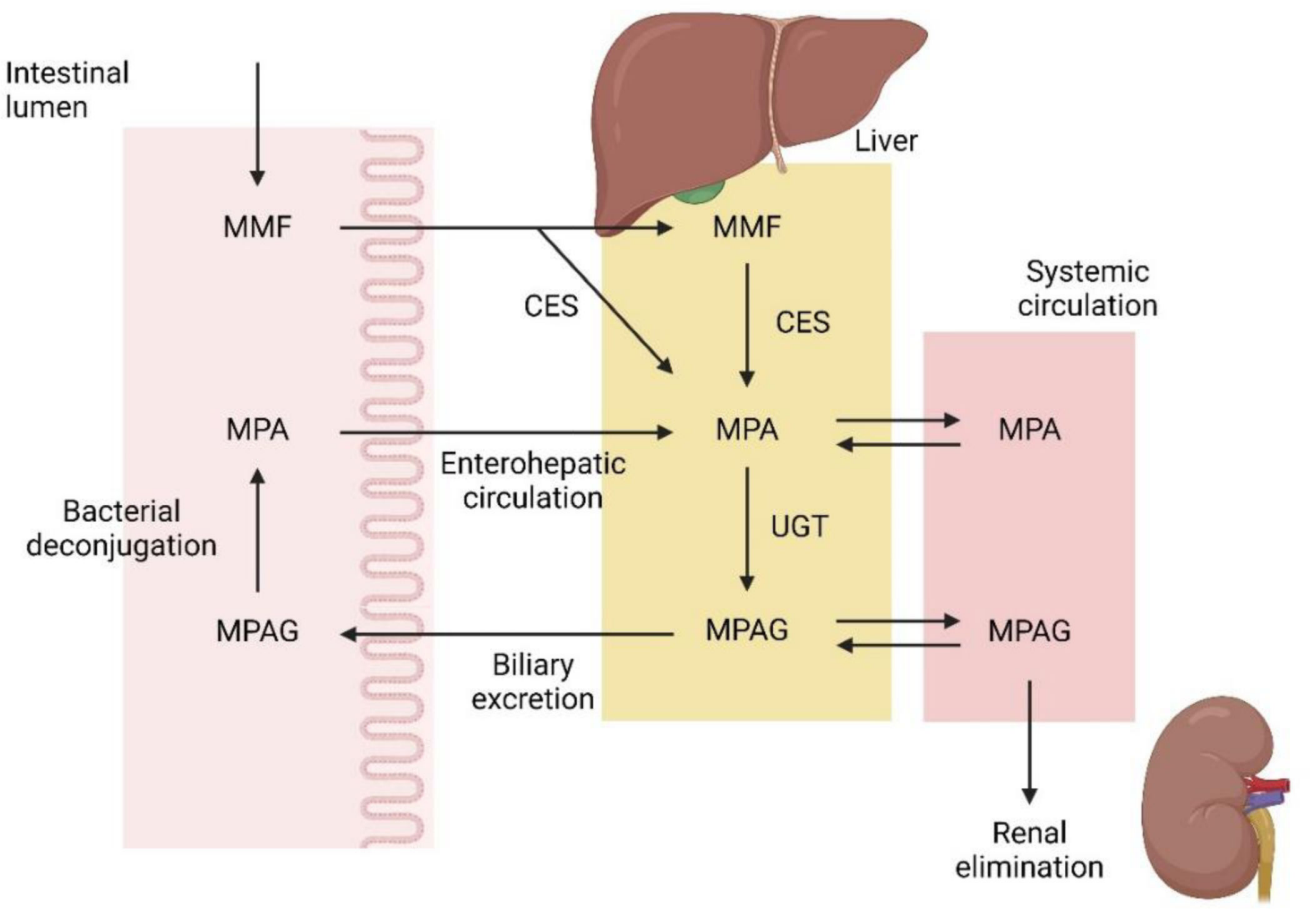
Pharmacokinetic pathway of MMF and MPA. Note: MMF-mycophenolate mofetil; MPA-mycophenolic acid; MPAG-MPA-glucuronide; CES-carboxylesterases; UGT-uridine 5’-diphospho-glucuronosyltransferases.

MMF, an ester prodrug, is rapidly converted to the biologically active moiety mycophenolic acid (MPA, Figure 1). Exposure to MPA is associated with MMF’s immunosuppressive and anti-inflammatory effect and the patient’s clinical response. However, the PK of MPA is characterized by large inter-individual and intra-individual variability. Factors contributing to this variability include differences in albumin concentration, corticosteroid use, impaired renal function, altered hepatic function, and genetic polymorphisms in drug metabolizing enzymes and drug transporters. Our previous work and that of other researchers [12–15], have shown that there is only a weak correlation between the dose of MMF (either based on weight or BSA) and the area under the time-concentration curve of MPA (MPA-AUC_0–12_). The apparent disconnect between MMF dosing based on body weight or BSA and MPA exposure stems from MPA’s complex metabolism which is influenced by concurrent therapies, the subject’s pharmacogenetic make-up, and presence of systemic disease including liver and renal function, which have all been extensively investigated by our group [12,16–20]. However, it remains uncertain whether higher MPA exposure with MMF dosing based on PK is well-tolerated, safe, and produces better renal outcomes compared to MMF dosing based on BSA when used for the treatment of pediatric LN. This underscores the need for therapeutic drug monitoring to optimize the use of MMF in pediatric LN. In this current study, our group aims to yield high-quality evidence for the superiority of pharmacokinetically-guided precision dosing over BSA-based dosing of MMF in pediatric LN.

## METHODOLOGY

### Design

**Figure 2:**
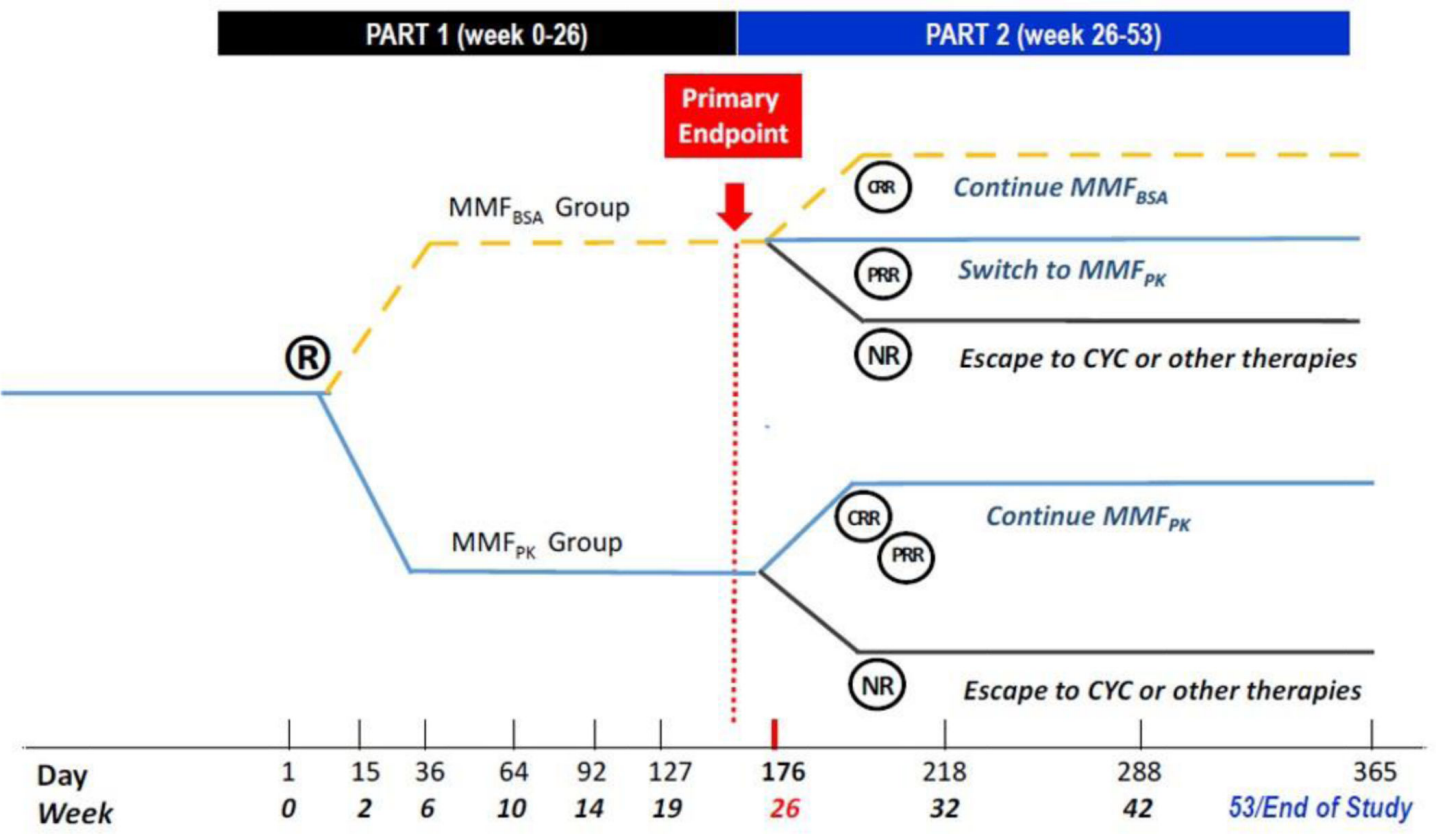
The PLUMM Study design. Note: R-randomization; MMF_BSA_-treatment arm of MMF dosed at 600 mg/m^2^ body surface area about 12 hours apart; MMF_PK_-treatment arm of MMF dosed twice daily to achieve an area under the concentration-time curve (AUC_0–12h_) of MPA ≥ 60–70 mg*h/L, CRR-complete renal response; PRR-partial renal response; NR-non-responder; CYC-cyclophosphamide.

This is a randomized double-blind active comparator clinical trial of pediatric subjects (8 to<18 years of age) with proliferative LN as per the International Society of Nephrology/ Renal Pathology Society (ISN/PRS) classification criteria [21]. All eligible subjects are randomized at baseline to one of 2 arms: the current standard of clinical care (MMF_BSA_) or pharmacokinetically-guided precision dosing (MMF_PK_). Subjects in the MMF_BSA_ arm who achieve PRR at week 26 will be switched to the MMF_PK_ arm. At the end of Part 1, subjects in the MMF_PK_ arm with CRR or PRR or MMF_BSA_ arm subjects with CRR will continue their respective treatment arms during Part 2 of the study. At week 26 (end of Part 1) treatment non-responders, i.e. subjects who did not achieve PRR at week 26, will be discontinued from active medication management in the study but will still be monitored for a total of 53 weeks. The MMF starting dose for both treatment arms will be the recommended MMF dose for pediatric LN at 600 mg/m^2^ twice daily, about 12 hours apart [22]. Only in the MMF_PK_ arm will the MMF dose be adjusted for a target MPA-AUC_0–12_ of 60 mg*h/L, with initial Bayesian PK-profiling performed after drug steady state is achieved. A schema of the study design is shown in Figure 2.

The study is overseen by a Centralized Coordinating Center (CCC), a study steering committee which includes a representative of the Lupus Foundation of America, and an independent Data Safety Committee that was selected by the National Institute of Arthritis and Musculoskeletal and Skin Diseases (NIAMS).

### Setting

The study will be conducted at 19 U.S. sites, all located at major pediatric academic centers. Site investigators are listed in ClinicalTrials.gov (NCT05538208). Site investigators are either board-certified pediatric rheumatologists or pediatric nephrologists.

### Population

Study participation is not restricted by patient sex, race, or ethnicity. Eligible patients fulfill all inclusion and no exclusion criteria. Given the potential use of immunosuppressants other than MMF for the treatment of proliferative LN, the decision to choose MMF is made as part of clinical care. The complete listing of the key eligibility criteria is provided in Table 1, while Supplementary Table 1 provides a full listing. Information about prohibited concurrent medications is shown in Supplementary Table 2.

### Outcome measures and safety and efficacy endpoints

The primary efficacy outcome of the study is the improvement of proliferative LN as measured by the presence of at least PRR at the end of Part 1 of the study (week 26). The major secondary outcome is the achievement of CRR at the end of Part 1 of the study. Other secondary outcomes are the achievement of CRR at the end of Part 2 of the study (week 53) and frequency of adverse events defined as grade 3 or higher during all parts of the study using the National Cancer Institute Common Terminology Criteria for Adverse Events.

Exploratory outcomes include: change in Renal Activity Index for Lupus (RAIL) score (baseline visit to week 53) [23,24], rate of LN flares, time to PRR and CRR during the study (baseline visit to week 53), mean time-adjusted score of the Systemic Lupus Erythematosus Disease Activity Index (SLEDAI) [25], considering extra-renal domain items during the study (baseline visit to week 53), cumulative dose of oral corticosteroids (CS; in prednisone-equivalents) and cumulative exposure to intravenous CS during the study, frequency of patients fulfilling discontinuation criteria during the study, change in health-related quality of life as measured by the Pediatric Quality of Life Inventory (PedsQL) Generic Core Scale [26], during the study, percentage of patients with intake of ≥ 80% of prescribed MMF doses as per blister-pack count during the study, and presence of MPA-levels<0.1 mg/L from random testing or in-clinic testing during the study. The MPA primary and secondary outcome evaluations will be performed by independent blinded evaluators at the CCC, according to validated criteria. Outcome definitions are provided in Table 2.

### Randomization

Eligible subjects enrolled in the study will be randomized (1:1) at baseline to receive either MMF_PK_ or MMF_BSA_. Randomization will be stratified by LN class (class 3 or 3/5 ***vs.*** class 4 or 4/5) as per the ISN/RPS and extrarenal manifestations of cSLE (extrarenal SLEDAI score<10 ***vs.*** ≥ 10). Race (white or non-white) and belimumab treatment (yes/no), as well as other important factors which have a known association with long-term outcomes of LN will be considered as potential confounders during the analyses. We will use variable block sizes to ensure balance and to minimize risk for unmasking. Thus, we will be using block sizes of 4 and 2 in random order.

### Blinding

Subjects and research personnel at the CCC and sites will remain blinded to the study intervention (MMF_PK_ or MMF_BSA_) until the end of the study. The following team members will remain unblinded: research pharmacist, unblinded study physicians, and the PK analysis team. During the study, masking will be maintained by using placebo capsules which have the same appearance as MMF. Genentech will supply MMF to the central pharmacy at Cincinnati Children’s Hospital Medical Center (CCHMC) Investigational Drug Service (IDS).

MMF tablets (500 mg/tab) and/or MMF capsules (250 mg/cap) and/or identical placebo capsules will be used to ensure double-blinding of the study treatment. Over-encapsulated MMF 250 mg and placebo capsules will be prepared by the IDS. Blister packs containing combinations of MMF 500 mg tablets, over-encapsulated MMF 250 mg, and/or placebo capsules are produced by the IDS to reflect the desired MMF dosage, based on the treatment arm. Each subject is instructed to take medication in the morning and evening from the appropriately labelled blister card containing 10 medication blisters. The appearance of study medication is changed after every study visit and study month, regardless of the treatment arm. After the final data lock, the site investigators will be informed of the treatment allocation.

### Investigational and reference therapy

MMF in tablet and/or capsule form will be taken twice daily by mouth, approximately 12 hours apart. The investigational therapy will be individualized using MMF_PK_ or MMF_BSA_. Based on PK assessment (as detailed in the next section), MMF dosage of the MMF_PK_ arm will be adjusted in 250 mg increments to achieve a target MPA-AUC_0–12_>60–70 mg*h/L. For the MMF_BSA_ arm, the amount of MMF prescribed will be around 600 mg/m^2^ BSA per dose, up to the recommended maximum of 3 grams per day. The dosage of MMF will be kept at the level prescribed on day 1 unless the patient’s weight changes. Necessary dose adjustments will be assessed whenever there is a weight change that exceeds 10 lbs. (or 4.5 kg) since the last dose. We expect trough levels of MPA to be>1–3.5 mg/L in both treatment arms.

### Pharmacokinetic assessment and Bayesian estimation

The study team developed a novel assay to measure MPA levels utilizing Volumetric Absorptive Microsampling (VAMS) devices from Mitra^®^ [27,28]. This will be used to measure MPA levels randomly or serially to assess MPA exposure and adherence to MMF. Capillary blood will be collected just prior to the next MMF intake (trough) and 20 minutes, 1 hour, and 3 hours after dose intake, which will then be sent to CCHMC for liquid chromatography tandem mass spectrometry (LC-MS/MS) drug assay and PK analysis. This method of PK assessment offers the convenient option of performing blood sampling for random MPA level measurement in the subject’s home setting.

The PLUMM study will use pharmacokinetic assessment at the following 5 patient visits during the study to adjust the dose of the MMF_PK_ arm subjects: baseline visit, week 3, week 10, week 26, and week 32. Dosing for this arm will be based on individual abbreviated AUC_0–12h_ estimates. These AUC_0–12h_ estimates will be generated with a well-established PK model-based Bayesian approach using MPA concentration results equivalent to plasma concentrations derived from capillary whole blood concentration results.

Two prospective studies were conducted at CCHMC and the University of Cincinnati (Dr. Rita Alloway, PI; CCHMC IRB: 2022–0416) in preparation for the PLUMM study. These studies were used to validate the methodology of the PLUMM study using the VAMS device for PK-profiling and to ensure the study team can reliably convert capillary whole blood concentrations (as obtained with the Mitra VAMS device) to plasma concentrations. Individual PK parameters and dose to achieve the target AUC_0–12h_ will be estimated with Bayesian estimation using clinical precision dosing software MwPharm++ (Mediware, Prague, Czech Republic). The Bayesian estimation method and the sparse sampling strategy (at 0 h, 20 min, 1 h, and 3 h) for MPA have been developed and validated in various patient populations including pediatric lupus patients by Dr. Pierre Marquet and colleagues at the Limoges University Hospital Laboratory of Pharmacology [29–32].

### Standardized steroid regimen

Corticosteroids (CS), mainly oral prednisone, are an integral part of treatment for patients with LN, but there is large variability of CS dosing among treating physicians [33]. Since CS have a strong anti-inflammatory effect that could influence the comparison of the two treatment arms and thereby affect the primary outcome of the study, site investigators are strongly encouraged to use the dose recommendations from the CCC which reflect a standardized steroid regimen as previously published by Chalhoub et al. [34].

Establishing a CS dosing algorithm was necessary to control the use of CS during the PLUMM study. Planning for the CS dosing was initiated with a retrospective chart review to document current use of CS at 15 sites. The chart review enabled the research team to build patient profiles to support consensus formation science using the Delphi methodology. The dosing parameters were developed based on the consensus ratings of 103 reviewing pediatric nephrologists and rheumatologists who rated 5056 patient profiles that outlined the disease course of LN patients. After the initial review, validation of the steroid dosing regimen was conducted with 60 raters on 1838 patient profiles. The resulting Standardized Steroid Regimen (SSR) has been published by Chalhoub et al. [34]. For the PLUMM study, the CCC will provide steroid dose recommendations based on data points from sites, using a calculator developed by one of the authors (BH).

### Phone application and other adherence tools

To alert subjects to random MPA testing and to remind of twice daily medication intake, the PLUMM phone application was developed which will be loaded onto the subjects’ device or parents’/caretaker’s device. We expect communication to occur primarily with the parent/caretaker. Random MPA measurements between study visits will be obtained if with suspected non-adherence or failure to log medication intake. An MPA level of<0.1 mg/L will be considered to reflect non-adherence.

### Sample size and power estimation

The PLUMM study is the largest clinical trial in pediatric LN, to the best of our knowledge, which is adequately powered. Sample size was determined from a combination of prevalence data and precedents in the literature [13,14,35–43]. A sample size of 45 subjects per group with proliferative LN is anticipated to provide a power of 80% for the primary aim of detecting a significant difference in the rate of PRR between the MMF_PK_ and MMF_BSA_ arms at the end of Part 1, at a 2-sided 5% significance level by a two-group Chi-square test, assuming the rate of PRR is 55% in MMF_BSA_ and 83% in MMF_PK_. The sample size of 90 will ensure 81% of power to detect a statistically significantly difference in the rate of CRR between the two study arms, assuming a smaller proportion of 14% and a larger proportion of 43%. Based on the above presented power analyses, we will plan to enroll 105 subjects, to allow for up to 14.3% attrition rate during Part 1 of the study.

### Statistical analysis

The primary outcome, superiority of MMF_PK_ over MMF_BSA_ for the percentage of subjects with clinical remission of LN (PRR, CRR) at the end of Part 1, will be tested using the analytical approach for the binomial outcome, such as Chi-square test, Fisher’s exact test, or a Bayesian approach with non-informative Beta prior for the binary probability parameter.

The choice of approach will be determined by the distribution of the data. Subjects who discontinue the treatment due to any reason will be considered as being non-responders. All secondary and exploratory efficacy outcomes will be analyzed by treatment group. For the binary secondary or exploratory outcomes, the analytical approach for the binary outcome, as used for the primary analysis, will be performed.

For the continuous exploratory endpoints, including change from baseline in PROMIS scores or RAIL scores, a mixed-effect model with repeated measures will be applied. The Kaplan-Meier plots will be generated for the exploratory endpoints of time to CRR and time to PRR. Since no measures are collected from the discontinued subject, the analyses will take an Inverse Propensity Weighting (IPW) approach where the propensity for subjects to be discontinued from each arm are estimated using logistic regression modeling or the covariate balance propensity score method.

The IPW approach will create pseudo subject samples that represent the original randomized subject cohort at the beginning of the study, and therefore addresses the missing data issue due to subject discontinuation.

Descriptive/summary statistics for all endpoints, with 95% confidence interval for treatment difference, will be provided. Baseline demographic characteristics, primary and major secondary outcomes will also be summarized overall and by age (<12 years ***vs.*** ≥12 years), gender and race (white ***vs.*** non-white). Safety analysis will be performed on all subjects who received at least one dose of study drug. Safety data will be subject to clinical review and summarized by appropriate descriptive statistics.

## DISCUSSION

The safety and efficacy of MMF in the treatment of LN have been tested in adults and in some pediatric subjects. However, data in both adults and children suggest that MMF_BSA_ does not reliably correlate with exposure to and subsequent immunological and clinical response to its biologically active metabolite, MPA [44]. Because of this weak correlation between MMF_BSA_ and MPA exposure, therapeutic drug monitoring presents a potential approach to optimize the use of MMF in pediatric LN.

The PLUMM study will provide high-level evidence that MMF_PK_ yields higher renal remission rates compared with MMF_BSA_ when used in the treatment of proliferative LN in cSLE. Currently, there are no controlled studies which provide a blinded, controlled head-to-head comparison of MMF_PK_ and MMF_BSA_ in either adult or pediatric LN. To the best of our knowledge, the PLUMM study is the first treatment trial in pediatric LN which is adequately powered.

We will collect data and biospecimens from 105 well-characterized pediatric LN patients, which will offer an unprecedented potential for collaborative ancillary studies. This will also allow for biobanking of DNA, RNA, plasma, and urine which will be used for supplementary studies of the pharmacogenetics (PG) of MPA. Future supplementary studies on MPA PG and the relationship of MPA with the pharmacology of hydroxychloroquine are anticipated to be successful, given the breadth of this study.

Additional strengths of the design of this study include a thorough evaluation of the pharmacokinetics of MMF in pediatric LN patients who are on concurrent medications, additional validation of a novel LN biomarker panel, and the testing of a novel SSR [34].

Notably, the SSR has been specifically developed for the PLUMM study in an international effort that used state-of-the art consensus methodology, statistical modeling, and true disease courses of children with LN. In brief, the SSR considers the time since LN diagnosis, the course of LN and extrarenal SLE, as well as the prior CS dosage since the preceding clinical assessment of the subject to suggest the dose of oral prednisone or IV methylprednisolone. Standardization of CS use is important, as CS are potent effect modifiers that require careful dosing in any randomized clinical trial of an inflammatory disease and has been identified to be one of the significant factors impacting the success of clinical trials in lupus [45,46]. This SSR is anticipated to manage and reduce variability of CS dosage using international consensus and real-life cSLE patient data.

The study design has some limitations. The feasibility of recruitment may be affected by the need in some patients for therapy with IV cyclophosphamide and/or B-cell depletion therapy (rituximab or belimumab). If such is deemed necessary for the treatment of a patient leading to their discontinuation of the study, their clinical outcome will still be monitored longitudinally. Concurrent use of calcineurin inhibitors may also affect MPA PK, so a subanalysis may be performed in those patients receiving this. Given the complex clinical course of cSLE patients, variability in CS dosing may persist despite SSR use, leading to potential deviation from the proposed standardized steroid regimen. If this was to be the case, then we will correct analyses for variation in CS dose and consider Bayesian analysis with causal interference strategies to correct for the CS effect. Since high doses of CS decrease exposure to MPA, we will repeat MPA PK measurement if a patient’s CS dose has changed by>50% or>20 mg since the last MPA PK profile. MPA PK may also be affected by concurrent medications. However, these will be kept stable during the study, other than CS.

## CONCLUSION

The PLUMM study is the first study of its kind to conduct a head-to-head comparison of pharmacokinetically-guided precision-dosing of MMF versus MMF dosed on body surface area, with respect to the achievement of renal disease remission in pediatric proliferative lupus nephritis. This study will yield high-quality evidence that will provide a foundation for leveraging therapeutic drug monitoring of MMF, thus leading to improved clinical outcomes in pediatric lupus nephritis.

## Supplementary Material

Supplementary

## Figures and Tables

**Table 1:** Key eligibility criteria.

Key inclusion criteria	Key exclusion criteria
1. Age 8-<18 years at the time of enrollment	1. Presence of features (from SLE or other chronic disease) that a-priori suggests that the subject benefits from other therapies than that suggested or allowable by the study protocol.
2. New diagnosis with proliferative LN as per the ISN/RPS classification criteria [21], based on kidney biopsy done within 60 days of enrollment. For study inclusion, the kidney biopsy needs to be newly interpreted as one of the following classes: class 3, class 3/5, class 4, or class 4/5	2. History of significant kidney disease prior to the diagnosis of SLE
3. Diagnosis with cSLE [47,48], per the classification criteria of the ACR [49]/EULAR [50] (i.e., SLE with diagnosis prior to or at age 18 years)	3. Estimated GFR <40 mL/min/1.73 m^2^ using the modified Schwartz equation [55].
4. Tolerates MMF as per the treating physician	4. Need for renal replacement therapy at the time of enrollment
5. SLEDAI-R [25,51–54], score>0	5. CYC within 12 weeks of enrollment
	6. Anti-CD20 monoclonal antibody treatment within 6 months, except if CD19/20+ counts are normal by flow cytometry analysis)
	7. Specific blood dyscrasias

**Note:** LN-lupus nephritis; ISN/RPS-International Society of Nephrology/Renal Pathology Society; cSLE-childhood-onset systemic lupus erythematosus; ACR-American College of Rheumatology; EULAR-European League Against Rheumatism; MMF-mycophenolate mofetil; SLEDAI-R-renal domain score of Systemic Lupus Erythematosus Disease Activity Index; GFR-glomerular filtration rate; CYC-cyclophosphamide.

**Table 2:** Definition of key efficacy measures.

Outcome measure	Definition	References
Partial renal remission	PRR is defined as relevant improvement of 2 LN-RV* with the remaining LN-RV being at least stable.	[22,34]
Complete renal remission	CRR is the presence of all 3 LN-RVs within normal range.	[22,34]
Lupus nephritis flare	A LN flare is the reproducible+ presence of 1 or more of the following (a-d) that is at least partially due to renal inflammation from LN per the treating physician. a. Newly abnormal GFR plus increase in hematuria by at least 2 categories (normal: 0 to 5 RBC/HPF (normal), 6 to 10 RBC/HPF, 11 to 25 RBC/HPF, 26 to 50 RBC/HPF, >50 RBC/HPF) OR new gross hematuria (>50 RBC/HPF). b. Abnormal GFR that decreased by >10% plus increase in hematuria by at least 2 categories (normal: 0 to 5 RBC/HPF (normal), 6 to 10 RBC/HPF, 11 to 25 RBC/HPF, 26 to 50 RBC/HPF, >50 RBC/HPF) OR new gross hematuria (>50 RBC/HPF). c. Persistent increase of UPCR to >0.5, after CRR d. Persistent doubling of UPCR with values >1.0, after PRR	[22,34]

**Note:** PRR-partial renal response; LN-RV-lupus nephritis core response variables; CRR-complete renal response; GFR-glomerular filtration rate; RBC/HPF-red blood cell per high-power field; UPCR-urine protein to creatinine ratio.

* LN-RVs are the upper limit of normal (ULN) or lower limit of normal (LLN) of the following:

Urine protein to creatinine ratio (ULN: 0.2), estimated GFR (LLN: 95 ml/min/1.73m^2^) and glomerular hematuria (ULN:0)

+ Reproducibility means presence on >2 subsequent time points >1 week apart.
