# Supplementary material for: Efficacy and Safety of Pharmacokinetically-Driven Dosing of Mycophenolate Mofetil for the Treatment of Pediatric Proliferative Lupus Nephritis–A Double-Blind Placebo Controlled Clinical Trial (The Pediatric Lupus Nephritis Mycophenolate Mofetil Study)
Source: J Clin Trials. Author manuscript; Available in PMC 2024 Jul 19. (PMC11258879)
Supplement: Supplementary [file NIHMS1999268-supplement-Supplementary.pdf]

Journal of Clinical Trials
ISSN: 2167-0870
OPEN ACCESS Freely available online
Research Article

Efficacy and Safety of Pharmacokinetically-Driven Dosing of Mycophenolate Mofetil for the Treatment of Pediatric Proliferative Lupus Nephritis–A Double-Blind Placebo Controlled Clinical Trial (The Pediatric Lupus Nephritis Mycophenolate Mofetil Study)

Anna Carmela P Sagcal-Gironella[1,2], Angela Merritt[3], Tomoyuki Mizuno[4,5], Vikas R Dharnidharka[6], Joseph McDonald[7], Marietta DeGuzman[8], Dawn Wahezi[9], Beatrice Goilav[10], Karen Onel[11], Susan Kim[12], Ellen Cody[13], Eveline Y Wu[14], Laura Cannon[14], Kristen Hayward[15], Daryl M Okamura[16], Pooja N Patel[17], Larry A Greenbaum[18], Kelly A Rouster-Stevens[19], Jennifer C Cooper[20], Natasha M Ruth[21], Stacy Ardoin[22], Kathryn Cook[23], R Ezequiel Borgia[24], Aimee Hersh[25], Bin Huang[26], Prasad Devarajan[27], Hermine Brunner[3*]

[1]Division of Pediatric Rheumatology, Hackensack University Medical Center, Hackensack, New Jersey, USA; [2]Department of Pediatrics, Hackensack Meridian School of Medicine, Nutley, New Jersey, USA; [3]Division of Rheumatology, Cincinnati Children's Hospital Medical Center, Cincinnati, Ohio, USA; [4]Division of Translational and Clinical Pharmacology, Cincinnati Children's Hospital Medical Center, Cincinnati, Ohio, USA; [5]Department of Pediatrics, University of Cincinnati College of Medicine, Cincinnati, Ohio, USA; [6]Department of Pediatric Nephrology, Washington University School of Medicine, Saint Louis, Missouri, USA; [7]Department of Pediatrics, University of Chicago, Chicago, Illinois, USA; [8]Department of Pediatrics, Baylor College of Medicine, Houston, Texas, USA; [9]Department of Pediatric Rheumatology, Children's Hospital at Montefiore, Albert Einstein College of Medicine, Bronx, New York, USA; [10]Department of Pediatric Nephrology, The Children's Hospital at Montefiore, Albert Einstein College of Medicine, Bronx, New York, USA; [11]Department of Pediatric Rheumatology, Hospital for Special Surgery, Weill Cornell Medicine, New York, New York, USA; [12]Department of Rheumatology, University of California, San Francisco, California, USA; [13]Department of Pediatric Nephrology, Medical College of Wisconsin, Milwaukee, Wisconsin, USA; [14]Department of Pediatric Rheumatology, University of North Carolina, Chapel Hill, North Carolina, USA; [15]Department of Pediatric Rheumatology, University of Washington, Seattle, Washington, USA; [16]Department of Pediatric Nephrology, University of Washington, Seattle, Washington, USA; [17]Depatrment of Pediatric Rheumatology, Ann & Robert H. Lurie Children's Hospital of Chicago, Northwestern University, Chicago, Illinois, USA; [18]Department of Pediatric Nephrology, Emory University and Children's Healthcare of Atlanta, Atlanta, Georgia, USA; [19]Department of Pediatrics, Emory University and Children's Healthcare of Atlanta, Atlanta, Georgia, USA; [20]Department of Pediatric Rheumatology, University of Colorado, Denver, Colorado, USA; [21]Department of Pediatric Rheumatology, Medical University of South Carolina, Charleston, South Carolina, USA; [22]Abigail Wexner Research Institute, Nationwide Children's Hospital, Columbus, Ohio, USA; [23]Division of Rheumatology, Akron Children's, Akron, Ohio, USA; [24]Department of Pediatric Allergy, Immunology and Rheumatology, UH Rainbow Babies & Children's Hospital, Cleveland, Ohio, USA; [25]Department of Pediatrics, Immunology and Rheumatology, University of Utah, Salt Lake City, Utah, USA; [26]Department of Pediatrics, University of Cincinnati College of Medicine and Cincinnati Children's Hospital Medical Center, Cincinnati, Ohio, USA; [27]Department of Nephrology and Hypertension, Cincinnati Children's Hospital Medical Center, Cincinnati, Ohio, USA

Correspondence to: Hermine Brunner, Division of Rheumatology, Cincinnati Children's Hospital Medical Center, Cincinnati, Ohio, USA, E-mail: hermine.brunner@cchmc.org

Received: 11-Apr-2024, Manuscript No. JCTR-24-30734; **Editor assigned:** 15-Apr-2024, PreQC No. JCTR-24-30734(PQ); **Reviewed:** 29-Apr-2024, QC No. JCTR-24-30734; **Revised:** 06-May-2024, Manuscript No. JCTR-24-30734(R); **Published:** 13-May-2024, DOI: 10.35248/2167-0870.24.14.561.

Citation: Sagcal-Gironella ACP, Merritt A, Mizuno T, Dharnidharka VR, McDonald J, DeGuzman M, et al. (2024) Efficacy and Safety of Pharmacokinetically-Driven Dosing of Mycophenolate Mofetil for the Treatment of Pediatric Proliferative Lupus Nephritis–A Double-Blind Placebo Controlled Clinical Trial (The Pediatric Lupus Nephritis Mycophenolate Mofetil Study). J Clin Trials. 14:561.

Copyright: © 2024 Sagcal-Gironella ACP, et al. This is an open-access article distributed under the terms of the Creative Commons Attribution License, which permits unrestricted use, distribution, and reproduction in any medium, provided the original author and source are credited.

Supplementary Table 1: Complete list of inclusion and exclusion criteria.

Inclusion criteria	Exclusion criteria
Male or female aged 8 to <18 years of age	Perceived or stated inability to adhere to the study protocol
Must meet Classification Criteria for SLE as per the criteria of the American College of Rheumatology (ACR)/ European League Against Rheumatism	Hypersensitivity to MMF or any component of the drug product
Newly diagnosed with proliferative LN as per the International Society of Nephrology/Renal Pathology Society based on kidney biopsy done within 60 days prior to enrollment into the study<br>Subjects may have been previously diagnosed with other classes of LN. For study inclusion, the kidney biopsy must be newly interpreted as one of the following classes: Class 3, Class 3/5, Class 4, or Class 4/5.	Presence of features (from SLE or other chronic disease) that a-priori suggest that the subject benefits from other therapies than that suggested or allowable by the study protocol<br>These disease features include but are not limited to severe, progressive, or uncontrolled hepatic, hematologic, gastrointestinal, metabolic, endocrine, pulmonary, cardiac, or neurologic disease.
SLE Disease Activity Index Renal Domain (Renal SLEDAI, SLEDAI-R) score >0	History of other kidney disease besides LN or prior to the diagnosis of SLE
Treatment of LN with twice daily MMF as per the decision of the treating physician.<br>The subject will have taken MMF as prescribed by the treating physician for a minimum of 4 days (or 8 doses).	Need for renal replacement therapy within 2 weeks from Baseline visit<br>Subjects can have required short-term renal replacement therapy prior to baseline visit, for example, due to preceding acute kidney injury.
Subject tolerates MMF as per the treating physician's opinion	Infections:<br>• Untreated latent or active tuberculosis (TB);<br>• Chronic infections requiring treatment;<br>• A subject known to be infected with Human Immunodeficiency Virus (HIV), Hepatitis B;<br>• Any infection requiring hospitalization, parenteral antimicrobial therapy or judged to be opportunistic by the investigator within 4 weeks prior of Baseline visit;<br>• Any treated infections within 2 weeks of Baseline visit;<br>• History of infected joint prosthesis with prosthesis still in situ.
Able to swallow MMF tablets and capsules	Blood dyscrasias:<br>• Hemoglobin <8.5 g/dL or Hematocrit <22%;<br>• White Blood Cell count <2.6 x 10^9/L;<br>• Neutrophil count <1.2 x 10^9/L;<br>• Platelet count <100 x 10^9/L;<br>• Lymphocyte count <0.5 x 10^9/L.
If subject is treated with belimumab, must be intravenous or subcutaneous	Estimated glomerular filtration rate (GFR) <40 mL/min/1.73 m^2 calculated using the modified Schwartz equation [56]
Subjects who are willing and able to comply with scheduled visits, treatment plan, laboratory tests, and other study procedures	Aspartate aminotransferase (AST) or alanine aminotransferase (ALT) >1.5 times the upper limit of normal
Evidence of a personally signed and dated Informed Consent document and Assent document (as appropriate) indicating that the subject and a legally acceptable representative/ parent(s)/legal guardian has been informed of all pertinent aspects of the study	Vaccinated or exposed to a live or attenuated vaccine within the 4 weeks prior to baseline visit
Parent or legal guardian must have a smart phone available and able to support the PLUMM smart phone application	History or current symptoms suggestive of lymphoproliferative disorders (e.g., Epstein Barr Virus [EBV] related lymphoproliferative disorder, lymphoma, leukemia, myeloproliferative disorders, or multiple myeloma)
Must be able to complete study questionnaires in English or Spanish	Current malignancy or history of any malignancy except for adequate treated or excised basal cell or squamous cell or cervical cancer in situ
	Recent (within 4 weeks prior to baseline visit) significant trauma or major surgery
	Herbal supplements with pharmaceutical properties must be discontinued at least 1week prior to Baseline visit, unless there are sufficient data available regarding the duration of an herbal medication's pharmacokinetic and pharmacodynamic effects to allow a shorter or longer washout to be specified (e.g., 5 half-lives)
	Hydroxychloroquine exceeding 5mg/kg/day or started within 1 week prior to baseline visit

	Oral or intravenous cyclophosphamide must be discontinued 12 weeks prior to baseline visit
	Rituximab or other selective B lymphocyte depleting agents<br>• Must be discontinued 6 months prior to Baseline visit or CD19/20+ counts must be normal by FACS analysis
	Use of prohibited prescription medication within the specified time frame prior to baseline visit
	Participation in other studies involving investigational drug(s) within 4 weeks or 5 half-lives (whichever is longer) prior to baseline visit and/or during study participation
	Pregnant female subjects; breastfeeding female subjects; male subjects with partners currently pregnant; male subjects able to father children and female subjects of childbearing potential who are unwilling or unable to use two highly effective methods of contraception or remain abstinent for the duration of the study
	Other severe acute or chronic medical or psychiatric condition or laboratory abnormality that may increase the risk associated with study participation or investigational product administration or may interfere with the interpretation of study results and, in the judgment of the investigator, would make the subject inappropriate for entry into this study

Supplementary Table 2: Prohibited concurrent medications.

**Leflunomide (Arava®)**	**8 weeks (without an elimination procedure*)**
4 weeks (with an elimination procedure)	O
Anakinra (Kineret®), Enbrel (Etanercept®)	4 weeks
Canakinumab (Ilaris®)	18 weeks
Adalimumab (Humira®)	6 weeks
Infliximab (Remicade®)	8 weeks
Golimumab (Simponi TM)	10 weeks
Abatacept (Orencia®), Tocilizumab (Actemra®), Certolizumab pegol (Cimzia®)	12 weeks
Rituximab (Rituxan®) or other selective B-lymphocyte depleting agents (either marketed or investigational)	6 months (or sooner, if CD19/20+ count is normal by FACS analysis)
Cyclophosphamide IV or oral	3 months
**Prohibited investigational drugs**	
Investigational non-steroidal anti-inflammatory drugs (NSAIDs), including selective COX-2 inhibitors	4 weeks
**Other investigational drugs/experimental therapy**	6 months or 5 half-lives (whichever is longer)
